# Acute Alcohol Intake Affects Internal Additive Noise and the Perceptual Template in Visual Perception

**DOI:** 10.3389/fnins.2022.873671

**Published:** 2022-05-13

**Authors:** Pan Zhang, Yeshuo Guo, Yuxin Qiao, Nan Yan, Yajing Zhang, Weicong Ren, Shilei Zhang, Di Wu

**Affiliations:** ^1^Department of Psychology, Hebei Normal University, Shijiazhuang, China; ^2^Huihua College, Hebei Normal University, Shijiazhuang, China; ^3^Military Medical Psychology School, Air Force Medical University, Xi’an, China

**Keywords:** alcohol, contrast sensitivity, external noise, perceptual template, spatial frequency

## Abstract

A reduction in visual performance is among the serious consequences of acute alcohol intake. Contrast sensitivity (CS) is a fundamental feature of visual function. Here, we investigated the negative effect of a moderate dose of alcohol on CS across a wide range of spatial frequencies and with multiple levels of external noise and clarified the corresponding mechanisms in the context of a perceptual template model (PTM). To avoid the effect of alcohol washout, a quick contrast sensitivity function (qCSF) method was used to assess the visual performance of subjects before and 30 min after a moderate dose of alcohol intake. We found that (1) CS was significantly disrupted by acute alcohol intake; (2) alcohol-induced CS loss was dependent on spatial frequency and external noise; and (3) increased internal additive noise and an impaired perceptual template accounted for alcohol-induced CS loss. These results help us better understand the negative effect of alcohol consumption and provide guidance for driver safety studies.

## Introduction

The consumption of alcoholic beverages is very popular worldwide. However, drunk driving is of great concern and is seriously penalized by governments due to its dangers (Taxman and Piquero, 1998). For example, a study examined 1,397 patients treated for motor vehicle injuries and found that alcohol-related impairment was strongly related to greater injury in non-admitted patients, even when crash severity was taken into account (Waller et al., 1989). In basic studies, it has been widely demonstrated that eye movements (Watten and Lie, 1997; Harvey, 2014; Tyson et al., 2021), basic perception (Wang et al., 2021), cognition (Silva et al., 2017; Zhang et al., 2020), emotion (Eastwood et al., 2020), and motor skills (Brumback et al., 2007; Petrović et al., 2017) are disrupted by alcohol intake and that the extent of these disruptions depends on the dose of alcohol. Negative effects have been observed even when subjects have low-to-moderate blood alcohol concentration (BAC) (Eckardt et al., 1998; Abroms et al., 2015).

Contrast sensitivity (CS), as a basic function of human vision, refers to the ability to distinguish a target from the background (Pelli and Bex, 2013). Although several studies have confirmed the negative effect of acute alcohol intake on CS (Zulauf et al., 1988; Andre et al., 1994; Nicholson et al., 1995; Cavalcanti-Galdino et al., 2014), these studies had several limitations. First, there were issues with the accuracy of the algorithm used to measure the contrast threshold measurement. For example, Zulauf et al. (1988) measured CS by incorporating an adjustment from before to after subjects drank alcohol. Specifically, the grating contrast was manually increased until the subject could identify the direction of the grating. However, the contrast threshold may have been overvalued due to adaptation. Second, the literature has not fully addressed the influence of acute alcohol intake on the contrast sensitivity function (CSF), which is typically assessed over a wide range of spatial frequencies (e.g., from 0.5 to 20 cycles per degree, cpd) to provide an exhaustive characterization of human vision (Chen et al., 2014). Numerous studies have found that various clinical conditions change CSF patterns. For instance, CSF deficits associated with amblyopia mainly occur at high spatial frequencies (Howell et al., 1983), and patients with dyslexia have problems processing targets at low spatial frequencies (Stuart et al., 2001). However, the literature usually evaluates alcohol-induced CS loss over only a narrow range of spatial frequencies. For example, Nicholson et al. (1995) measured CS at only three spatial frequencies (e.g., 1.5, 6, and 12 cpd).

To avoid contrast adaptation effects, some studies applied staircase methods (e.g., three-down/one-up). For example, Cavalcanti-Galdino et al. (2014) assessed CS for gratings and angular frequency stimuli in adult subjects after acute alcohol intake. The spatial frequencies for sine-wave gratings were 0.25, 1.25, 2.5, 4, 10, and 20 cpd. In contrast, the angular frequencies for angular frequency stimuli were 1, 2, 4, 24, 48, and 96 cycles per 360 degrees. However, a full CSF assessment (e.g., distributed over 10 spatial frequencies) with the three-down/one-up method requires 1,000–1,200 trials and may take 80–100 min; the peak effect of acute alcohol intake may not last this long. Thus, it is necessary to develop a quick CSF (qCSF) test with both high precision and efficiency. Fortunately, a qCSF method was developed with a Bayesian adaptive framework (Lesmes et al., 2010). This algorithm exhibited excellent precision and accuracy in CSF measurements and thus has been widely used to investigate factors affecting visual function, such as the investigation of retinal position (Rosén et al., 2014) and arousal (Lee et al., 2014). In addition, this algorithm is very helpful for identifying patients with visual deficits, e.g., amblyopia (Hou et al., 2010), glaucoma (Shakarchi et al., 2019), myopia (Chen et al., 2019), and those due to aging (Yan et al., 2020). The present work fully investigated the negative effects of acute alcohol intake on CSF with the qCSF procedure.

In addition to spatial frequencies, the CSF is also modulated by external noise. Some studies have demonstrated that the CSF can be flattened after adding external noise with high contrast to the to-be-detected gratings (Xu et al., 2006; McAnany and Alexander, 2010). This indicates that the gain of the visual system does not simply determine the CSF (Rovamo et al., 1992; McAnany and Alexander, 2010). In real life, weather conditions such as fog or snow limit the ability of drivers to detect a car or pedestrian on the road. Thus, the current work further examines the effects of alcohol on CSF in contexts with different external noise levels.

The perceptual template model (PTM) can explain the changes in visual perception through three mechanisms (Dosher and Lu, 1999). The first is internal additive noise, which amplifies both the signal and the noise from input stimuli. The second is called the perceptual template, which helps the visual system exclude external noise. The third is internal multiplicative noise, which refers to contrast-gain control properties. The PTM has been successfully used to determine the advantages of binocular CS over monocular CS (Zhang et al., 2021) and the improvements induced by perceptual learning and reward (Dosher and Lu, 1999; Zhang et al., 2018). Thus, a combination of external noises and the PTM provides an ideal method to clarify the mechanisms underlying the effects of alcohol on visual function.

Therefore, our specific aims included (1) fully investigating the negative effects of acute alcohol intake on CS across multiple spatial frequencies and external noise levels and (2) determining the corresponding mechanisms within the PTM framework. Since higher GABA levels are correlated with reduced visual performance (e.g., contrast discrimination) (Schallmo et al., 2020), a decline in CS at multiple spatial frequencies was expected. In addition, alcohol-induced CS deficits may be more profound in visually noisy environments.

## Method

### Participants

In total, nine participants (all over 23 years old) were recruited from the college. All participation was voluntary, as indicated by signing clear consent forms. Before the experiment, they were tested to ensure that their visual acuity (VA) (or corrected VA) was ≥1.0 (decimal notation) and assessed for the no history of ocular, somatic, neurological, or psychiatric disease, and drinking within the 7 previous days. In addition, subjects should have no history of substance dependence, such as caffeine, alcohol, cigarettes, and drugs. This work was authorized by the Ethics Committee of Huihua College of Hebei Normal University and followed the Declaration of Helsinki.

### Apparatus

Stimuli were produced by the Psychophysics Toolbox (Pelli, 1997) and presented on a cathode-ray tube (CRT) monitor. The background luminance of the display was 35.9 cd/m^2^ with an 85-Hz frame rate and 1,024 × 768 resolution. To produce 14 bits of gray level, a special circuit was used (Li et al., 2003). The viewing distance was 183 cm. The subjects completed the experiment binocularly.

### Stimuli

Vertical gratings at 10 spatial frequencies (0.5, 0.67, 1, 1.33, 2, 2.67, 4, 5.33, 8, and 16 cpd) were target stimuli. External noise images served as distractors. The multiplication of the sizes (in degrees) and spatial frequencies (in cpd) of the stimuli was fixed at 3. Thus, the gratings were always displayed for three cycles. To blur the edge of each grating, they were covered with truncated Gaussian envelopes. The sizes of the noise images were the same as those of the gratings (before placement of the envelope). To match the spectrum energy generated by the noise images and the gratings at each spatial frequency condition, each noise image consisted of the same number of noise elements (15 × 15).

### Procedures

**FIGURE 1:**
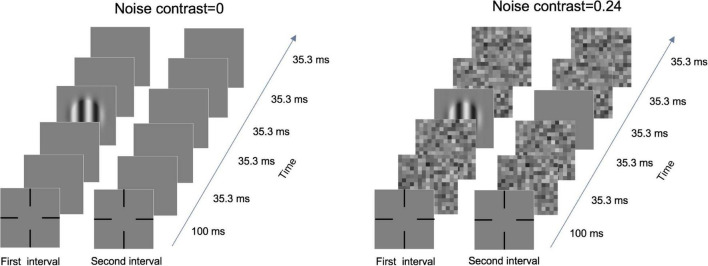
Illustration of a typical trial under zero- (left) and high- (right) noise conditions.

The qCSF procedure was used to measure the CSF (Figure 1). Each trial had two intervals, which sandwiched a 500-ms blank. A grating was presented in the first or second interval. Each interval included five 35.3-ms images (frames). The participants were required to identify the interval containing the grating by using a gamepad. In the noise condition, each interval consisted of one blank or grating image and four noise images. Under the zero-noise condition, blank images replaced the noise images. In each trial, noise images of the same size as the signal grating (before the Gaussian patch) were randomly sampled from the normal distribution, where the standard deviation (SD) was 0 (zero noise), 0.12 (low noise), or 0.24 (high noise), but the mean was always zero. The noise images in different spatial frequency conditions had the same number of noise elements. Regardless of accuracy, each response was followed by a brief beep. The three noise levels were randomly mixed across trials. Each noise condition consisted of 50 trials.

### Design

The experiment was divided into three stages: (a) pre-test of the binocular VA and CSF, (b) alcohol consumption, and (c) post-test of the binocular VA and CSF. During the pre-test, subjects’ VA and CSF were assessed by the E-chart and the qCSF procedure, respectively. In addition, the subjects first completed the VA test in an illuminated room, and then the light was turned off. After 5 min of dark adaptation, CSF measurements were started, which took less than 15 min. After they were completed, the light was turned back on.

To simulate real-life alcohol consumption, Chinese liquor (Quanxing Baijiu, China) with a 52% alcohol content was chosen. The subjects were asked to finish the 100-ml drink within a 15-min period. After 20 min, the blood alcohol concentration (BAC) of the subjects had reached 0.822 ± 0.089 mg/ml, and the subjects’ VA and CSF were assessed again. Before the experiment, all subjects were asked to eat some light food, and the experiment always began approximately 2 h after breakfast or lunch.

To better evaluate the effect of alcohol, the area under the log contrast sensitivity function (AULCSF, in log_10_ units) was computed to quantify the CS over 10 spatial frequencies (Zhang et al., 2018, 2021; Wu et al., 2020, 2021a,b). The AULCSF was calculated for each external noise condition.

#### Perceptual Template Model Analysis

Based on the PTM, we calculated a subject’s performance with the following equation:


(1) $$d^{\prime }=\frac{{\left(\beta c\right)}^{\gamma }}{\sqrt{{\left(A_{f}N_{ext}\right)}^{2\gamma }+A_{m}^{2}N_{mul}^{2}\left({\left(\beta c\right)}^{2r}+{\left(A_{f}N_{ext}\right)}^{2\gamma }\right)+{\left(A_{a}N_{add}\right)}^{2}}},$$

where *d*′ represents the performance; the signal contrast is expressed by *c*; the equivalent internal additive and multiplicative noise are denoted by *N*_*add*_ and *N*_*mul*_, respectively; *N*_*ext*_ is the contrast of external noise; γ indicates the system’s non-linearity; and β is the perceptual template gain. *A*_*a*_, *A*_*f*_, and *A*_*m*_ were used to simulate the influence of alcohol intake on *N*_*add*_, *N*_*ext*_, and *N*_*mul*_, respectively. Thus, before alcohol intake, the values of *A*_*a*_, *A*_*f*_, and *A*_*m*_ were fixed at 1. As the slopes of psychometric functions were unchanged before and after alcohol intake, the multiplicative noise was set as a constant (details are listed in Supplementary Material). Thus, *A*_*m*_ was deleted from Eq. 1 (Xu et al., 2006). In addition, *N_*add*_* and β varied across different spatial frequency conditions, but *N_*mul*_* and γ did not vary at different spatial frequency conditions. In brief, two possible mechanisms were deemed to explain the effects of alcohol on visual perception: higher internal additive noise and disrupted perceptual template (ability to filter external noise).

There were four models. In the full model, alcohol intake was considered to increase internal additive noise and disrupt the perceptual template. The reduced model 1 hypothesized that alcohol intake only increased internal additive noise, and the reduced model 2 hypothesized that alcohol intake only disrupted the perceptual template. None of the parameters changed in the most reduced model.

The goodness of fit (*r*^2^) was used to index the model fit:


(2) $$r^{2}=1-\frac{\sum {\left(y_{i}-{\hat{y}}_{i}\right)}^{2}}{\sum {\left(y_{i}-\bar{y}\right)}^{2}},$$

where the predicted and original data are indicated by ${\hat{y}}_{i}$ and *y_i_*, respectively; the mean of all original data is denoted by $\bar{y}$.

To investigate which model was best, an *F* test was used (Xu et al., 2006; Huang et al., 2009, 2010, 2011; Bejjanki et al., 2014; Zhang et al., 2018):


(3) $$\text{F}\left(k_{full}-k_{reduced},N-k_{full}\right)=\frac{\left(r_{full}^{2}-r_{reduced}^{2}\right)/\left(k_{full}-k_{reduced}\right)}{\left(1-r_{full}^{2}\right)/\left(N-k_{full}\right)},$$

where the number of parameters of each model is indicated by *k*, and the number of data points is expressed by *N*. The best model should be better than all its reduced models and comparable to the full model base on *r*^2^.

## Results

**FIGURE 2:**
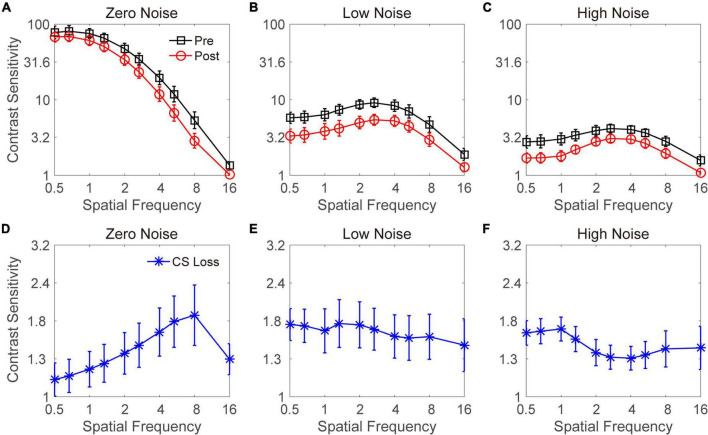
Contrast sensitivity functions at zero- **(A)**, low- **(B)**, and high- **(C)** noise levels. Black lines with squares and red lines with circles indicate contrast sensitivity before and after acute alcohol intake, respectively. Blue lines with asterisks indicate contrast sensitivity loss at zero- **(D)**, low- **(E)**, and high- **(F)** noise levels. Data were averaged across subjects. Error bars denote standard error (SE).

A paired *t*-test showed that alcohol intake did not change VA [*t*(17) = 1.449, *p* = 0.185]. The curves in Figure 2 show CS at three noise levels before and after alcohol intake. The pattern shows that acute alcohol consumption reduced CS in the three noise conditions. We performed a repeated-measures analysis of variance (ANOVA) on CS in the zero-noise condition with time point (before vs. after) and spatial frequency (from 0.5 to 16 cpd) as two within-subject variables. Significant main effects of time point and spatial frequency and an interaction effect were found [*F*(1,8) = 5.253, *p* = 0.051; *F*(9,27) = 206.857, *p* < 0.001; *F*(9,72) = 7.277, *p* < 0.001, respectively]. The least significant difference (LSD) test revealed that acute alcohol intake significantly (or marginally significantly) decreased CS at 2–16 cpd (all *p* < 0.070). Although there were no significant differences at low spatial frequencies (e.g., 0.5, 0.67, 1, and 1.33 cpd), a trend toward decreased CS was observed.

A repeated-measures ANOVA was conducted on CS in the low-noise condition with time point (before vs. after) and spatial frequency (from 0.5 to 16 cpd) as two within-subject factors. There were significant main effects of time point and spatial frequency [*F*(1,8) = 21.040, *p* = 0.002; *F*(9,27) = 12.443, *p* < 0.001, respectively]. In contrast, the interaction between time point and spatial frequency failed to reach significance [*F*(9,72) = 0.177, *p* = 0.996]. These findings indicated that acute alcohol intake significantly disrupted CS at all spatial frequencies when noise images with low intensity were added. Then, the same analysis was applied to the CS in the high-noise condition. Significant main effects of time point and spatial frequency were observed [*F*(1,8) = 23.405, *p* = 0.001; *F*(9,27) = 8.678, *p* < 0.001, respectively]. In contrast, no significant interaction effect was observed between time point and spatial frequency [*F*(9,72) = 1.256, *p* = 0.276]. These findings suggest that acute alcohol intake significantly disrupts CS at all spatial frequencies when high noise was present. In summary, when external noise was present, CS at all spatial frequencies was impaired by acute alcohol intake; in contrast, when external noise was absent, CS reductions after alcohol drinking were mainly at intermediate and high spatial frequencies.

To explore the decrease in CS under each of the spatial frequency and external noise conditions, CS loss was computed by subtracting the CS in the post-test from that in the pre-test. The CS losses at zero-, low-, and high-noise levels are plotted in Figures 2D–F, respectively. A repeated-measures ANOVA was performed on the CS loss with the external noise level and spatial frequency as two within-subject variables. The main effects of external noise level and spatial frequency were not significant [*F*(2,16) = 0.913, *p* = 0.421; *F*(9,72) = 0.367, *p* = 0.947, respectively]. However, the interaction effect between them was significant [*F*(18,144) = 2.051, *p* = 0.010]. The LSD tests revealed that when noise was absent, the CS loss at low spatial frequencies (e.g., 0.5, 0.67, and 1 cpd) was comparable (all *p* > 0.1) but significantly (or marginally) smaller than those at the middle (1.33, 2, and 2.67 cpd) and high (4, 5.33, and 8 cpd) spatial frequencies (all *p* < 0.078), except for the CS loss at 0.5 and 1.33 cpd, which was comparable (*p* = 0.134). The CS loss at 16 cpd was not significantly different from that at any other spatial frequencies (all *p* > 0.1). As shown in Figure 2D, we observed a clear pattern of higher spatial frequencies with larger CS losses, except 16 cpd. When stimuli were presented with low or high noise, the CS losses at each spatial frequency were not significantly different from each other, although the data in Figures 2E,F exhibited a larger CS loss at lower spatial frequencies.

**FIGURE 3:**
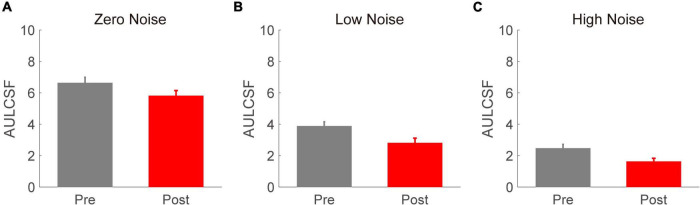
The area under the log contrast sensitivity function (AULCSF) (log_10_ units) values in zero- **(A)**, low- **(B)**, and high- **(C)** noise conditions. Gray and red bars denote the results before and after acute alcohol intake, respectively. Error bars denote SEs.

To better evaluate the effect of acute alcohol intake on the CSF, AULCSF was calculated for each time point and external noise condition (as shown in Figure 3). We conducted a repeated-measures ANOVA on AULCSF with time point (before vs. after) and external noise (zero, low, and high) as two within-subject variables. The main effects of time point and external noise were found to be significant [*F*(2,16) = 97.184, *p* < 0.001; *F*(1,8) = 17.248, *p* = 0.003, respectively]. In contrast, the interaction effect between time point and spatial frequency failed to reach significance [*F*(2,16) = 0.630, *p* = 0.545]. Then, another repeated-measures ANOVA was performed on the AULCSF reduction with external noise as a within-subject factor. We found a significant main effect of external noise [*F*(2,16) = 4.365, *p* = 0.031]. The LSD tests revealed that the AULCSF reduction in the zero- and low-noise conditions (3.341 ± 0.407 and 2.257 ± 0.300, respectively) was significantly smaller than that in the high-noise condition (3.819 ± 0.519). In addition, the AULCSF reductions at the zero- and low-noise levels were comparable. These findings indicate that the alcohol-induced AULCSF reduction was dependent on external noise, with larger reductions at higher external noise levels.

**FIGURE 4:**
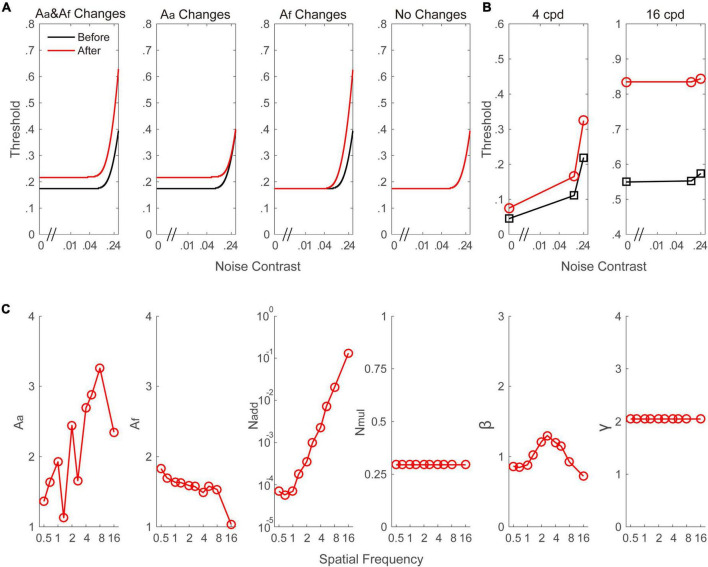
**(A)** A simulated signal contrast threshold as a function of the external noise level under different model assumptions. In the first column (*A*_*a*_ and *A*_*f*_ changes), alcohol intake increased the internal additive noise and impaired the perceptual template, leading to an increase in the threshold across all external noise contrast. In the second column (*A*_*a*_ changes), alcohol intake only increased the internal additive noise, leading to an increase in threshold in the region of low external noise contrast where performance is limited by internal additive noise. In the third column (*A*_*f*_ changes), alcohol intake only impaired the perceptual template, resulting in improved thresholds at high levels of external noise. In the fourth column (no changes), the curve is constant before and after alcohol intake. **(B)** Signal contrast threshold versus noise contrast curve at 4 and 16 cpd, respectively. The curves were drawn based on the parameters from the best-fitting model. **(C)** *A*_*a*_, *A*_*f*_, *N*_*add*_, *N*_*mul*_, and γ as a function of spatial frequencies from the best-fitting model. Black and red color indicate data from before and after alcohol intake, respectively.

To identify the mechanisms underlying the alcohol-induced CS reductions, the data averaged across individuals were fitted to the PTM. To better understand the quantitative hypotheses that our study examined, we plotted the signal threshold versus noise contrast (TvC, as shown in Figure 4A) curves that the PTM predicts if the visual performance is determined by a change in one or two of the parameters (*A*_*a*_, *A*_*f*_, or both). Thus, the three noise levels in the current were able to distinguish the different mechanisms in the PTM. The best-fitting model was defined as the one that produced a comparable *r*^2^ value with the full model but was constructed by the fewest free parameters. From the full to most reduced models, the *r*^2^ values were 97.3% (*A*_*a*_ and *A*_*f*_ change), 82.9% (*A*_*a*_ change), 89.1% (*A*_*f*_ change), and 74.8% (no change). We found that the full model was the best-fitting model because its *r*^2^ value was significantly better than those of any reduced model (all *p* < 0.001). To further confirm the finding, the Akaike information criterion (AIC) was computed. The AIC scores of the full model (*A*_*a*_ and *A*_*f*_ change), reduced model 1 (*A*_*a*_ change), reduced model 2 (*A*_*f*_ change), and most reduced model (no change) were 5.928, 6.594, 6.741, and 7.108, respectively. The model with the smaller AIC score was chosen as the best model. Thus, the full model was the best-fitting model in the current study. The predicted TvC curves at two typical frequencies (e.g., 4 and 16 cpd) were drawn in Figure 4B. The best-fitted parameters are plotted in Figure 4C. Average across spatial frequencies, the *A*_*a*_ and *A*_*f*_ were 2.133 ± 0.221 and 1.559 ± 0.065, respectively. In addition, as shown in Figure 4C, both parameters might be dependent on spatial frequency. To confirm this assumption, two Pearson correlation analyses were performed to investigate the relationship between spatial frequency and *A*_*a*_ as well as *A*_*f*_. *A*_*a*_ was positively correlated with the spatial frequency (*r* = 0.735, *p* = 0.015). In contrast, *A*_*f*_ was negatively correlated with the spatial frequency (*r* = –0.848, *p* = 0.002). These findings indicate that both mechanisms contributed to alcohol-induced CS loss, and these changes were strongly modulated by spatial frequency.

## Discussion

The current study explored the negative effects of acute alcohol intake on CS and investigated the mechanisms of perception alteration with the PTM framework. Alcohol-induced CS loss was observed and was strongly dependent on the external noise levels and spatial frequency conditions. In addition, acute alcohol intake amplified internal additive noise and deteriorated the perceptual template.

Alcohol-induced CS decreases have been examined in previous studies, but the current study utilized an improved experimental design. First, the CSF measurement in our study was highly precise and efficient due to the qCSF procedure. Unlike the traditional staircase method, which requires hours to assess the whole CSF at multiple spatial frequencies, the qCSF method is able to provide large amounts of information on CSF before the peak of the alcohol effect is washed out. This is very important because a previous study found that the BAC started to decline after approximately 70 min (Wang et al., 2018). This quicker method also prevents confusion between the effects of alcohol effect and those of fatigue. Second, we were able to collect the CSF at ten spatial frequencies. A clear pattern of alcohol-induced CS loss versus spatial frequency function was obtained. We found that the CS loss was mainly at middle and high spatial frequencies, and these findings shed light on driver safety.

The external noise intensity-dependent AULCSF reduction is another highlight of the current study. The AULCSF provides an overall assessment of CS, which has never been done before. We found that the AULCSF reduction was larger in high-noise conditions and was comparable in low- and zero-noise conditions. In addition, the AULCSF reduction was more profound at high spatial frequencies in the absence of external noise. In addition, we observed a weak trend of less AULCSF loss at high spatial frequencies when external noise was present. We hypothesize that alcohol intake suppressed the processing of gratings much more at high spatial frequencies. In contrast, when noise was present, both signal and noise were suppressed by alcohol; thus, the performance of contrast detection experienced a relative benefit (although the cost was still larger than the benefit) at high spatial frequencies. Additionally, the CSF curves of low and high noise conditions were band-pass filtered, but that of the zero-noise condition was low-pass filtered, which is consistent with previous studies (Chen et al., 2014; Zhang et al., 2021).

A widely accepted assumption is that the perceptual system is limited by an equivalent internal noise source whose amplitude is not modulated by the input (Barlow, 1956). This is an example of internal additive noise. Researchers often construct the simplest model for an observer with a noise-free linear amplification, an internal additive noise, and a decision process (Lu and Dosher, 1999). The internal additive noise reflects the degree of inefficiency exhibited by the perceptual system in visual processing. In the current study, we systematically increased the intensity of external noise to the signal grating and examined how the contrast sensitivity varied at different external noise levels in a contrast detection task. According to the PTM, alcohol-induced CS loss is explained by the higher internal noise and disrupted perceptual template. In addition, the changes resulting from these two factors were highly dependent on the spatial frequency. Specifically, the amplification of internal noise was more severe at higher spatial frequencies. This finding was expected because the CS loss at low spatial frequencies was much smaller than that at high spatial frequencies when external noise was absent. At the neurotransmitter level, the downregulation of GABAergic inhibition in the visual cortex may be the neural cause for internal additive noise elevation after alcohol intake. The function of a GABA-A receptor has been demonstrated to be enhanced by low-to-moderate dosages of alcohol (Harris et al., 1995). Thus, from a different perspective, the negative effect of alcohol would be amplified by GABA agonists but diluted by GABA antagonists (Lobo and Harris, 2008). The higher activity of the GABA-A receptor can increase the signal-to-noise ratio and reduce excitability.

In contrast, the disruption in the perceptual template was more severe at lower spatial frequencies. In functional magnetic resonance imaging (fMRI) research, acute alcohol intake increased spontaneous BOLD fluctuations in V1 (Esposito et al., 2010). Since the cells in V1 are each responsive to stimuli at a narrow band of spatial frequencies (De Valois and De Valois, 1988) and the signal (grating) was temporally sandwiched by noise images in the current study, some cells will be tuned to the spatial frequencies common to the grating and external noise, whereas others will be tuned to spatial frequencies that are only present in the external noise (Lu et al., 2011). After alcohol intake, the latter cells may not be strongly suppressed, resulting in a stronger BOLD response when the gratings are combined with mostly external noise. Future examination of this assumption is merited. Our approach also sheds light on the visual deficits in patients with alcohol addiction. We hypothesize that alcohol abuse may impair the perceptual template (the ability to exclude external noise or masks) and increase the internal additive noise (the degree of inefficiency) of the visual system.

Measuring the effects of alcohol intake on visual performance has important implications for driving safety. A static foveal VA test is widely used to assess the quality of vision in typical applicants for a driver’s license. However, in the current study, VA was not sensitive to the moderate dose of alcohol. We suggest that CS at multiple external noise levels should be taken into consideration when alcohol consumption policies are created.

## Data Availability Statement

The raw data supporting the conclusions of this article will be made available by the authors, without undue reservation.

## Ethics Statement

The studies involving human participants were reviewed and approved by Ethics Committee of Huihua College of Hebei Normal University. The patients/participants provided their written informed consent to participate in this study.

## Author Contributions

PZ, DW, and SZ designed the experiments and wrote the manuscript. PZ and SZ performed experiments and data analysis. YG, YQ, NY, YZ, and WR participated in data analysis. All authors discussed and commented on the manuscript.

## Conflict of Interest

The authors declare that the research was conducted in the absence of any commercial or financial relationships that could be construed as a potential conflict of interest.

## Publisher’s Note

All claims expressed in this article are solely those of the authors and do not necessarily represent those of their affiliated organizations, or those of the publisher, the editors and the reviewers. Any product that may be evaluated in this article, or claim that may be made by its manufacturer, is not guaranteed or endorsed by the publisher.
