# Supplementary material for: Acute Alcohol Intake Affects Internal Additive Noise and the Perceptual Template in Visual Perception
Source: Front Neurosci. 2022 May 13;16:873671. doi: 10.3389/fnins.2022.873671 (PMC9136069; doi:10.3389/fnins.2022.873671)
Supplement: Supplementary file 1 [file Data_Sheet_1.docx]

**Supplementary Information**

**Slope Check in Measurement of the Contrast Sensitivity Function**

At first, we need to obtain the slopes of the psychometric function before and after alcohol intake. The equation is as follows:

$P_{i,j}\left( x \right)=\gamma+\left( 1-\gamma-\lambda\right)\left( 1-exp\left( -{10}^{s\left( {log}_{10}\left( x \right)-{log}_{10}\left( \tau_{i,j} \right) \right)} \right) \right)$, Eq. S1

where the unique free parameter is *s*, which denotes the slope of psychometric function; *τ* is the contrast threshold at 80.3% correct performance level; guessing rate (*γ*) is 0.5 and lapse rate (*λ*) is 0.02; *P_i,j_*(*x*) is percent correct in the *i*^th^ spatial frequency and *j*^th^ external noise level. A t test was performed on the slopes with time point (before and after alcohol intake) as a within-subject variable. We found that slopes were unchanged after alcohol intake (t(8) = 9.61, *p* = 0.365),.

**Fitting the PTM**

The raw CSF data could derive one psychometric functions with six contrast level, corresponding to 60%, 70%, 78%, 84%, 90%, and 99% correct. Ten spatial frequencies, three external noise levels, and two time-point conditions produced 60 psychometric functions. Thus, a total of 360 data points were included in model fitting.
